# Dental pulp stem cell-derived exosomes suppress M1 macrophage polarization through the ROS-MAPK-NFκB P65 signaling pathway after spinal cord injury

**DOI:** 10.1186/s12951-022-01273-4

**Published:** 2022-02-02

**Authors:** Chao Liu, Fanqi Hu, Genlong Jiao, Yue Guo, Pan Zhou, Yuning Zhang, Zhen Zhang, Jing Yi, Yonggang You, Zhizhong Li, Hua Wang, Xuesong Zhang

**Affiliations:** 1 Department of Orthopaedics, The First Affiliated Hospital of Jinan University, Huangpu Avenue West Road, Guangzhou, People’s Republic of China; 2 Department of Orthopaedics, Chinese People’s Liberation Army General Hospital, Beijing, People’s Republic of China; 3 Beijing Institute of Radiation Medicine, Beijing, People’s Republic of China

## Abstract

Stem cell-derived exosomes have recently been regarded as potential drugs for treating spinal cord injury (SCI) by reducing reactive oxygen species (ROS) and suppressing M1 macrophage polarization. However, the roles of ROS and exosomes in the process of M1 macrophage polarization are not known. Herein, we demonstrated that ROS can induce M1 macrophage polarization and have a concentration-dependent effect. ROS can induce M1 macrophage polarization through the MAPK-NFκB P65 signaling pathway. Dental pulp stem cell (DPSC)-derived exosomes can reduce macrophage M1 polarization through the ROS-MAPK-NFκB P65 signaling pathway in treating SCI. This study suggested that DPSC-derived exosomes might be a potential drug for treating SCI. Disruption of the cycle between ROS and M1 macrophage polarization might also be a potential effective treatment by reducing secondary damage.

**Graphical Abstract:**

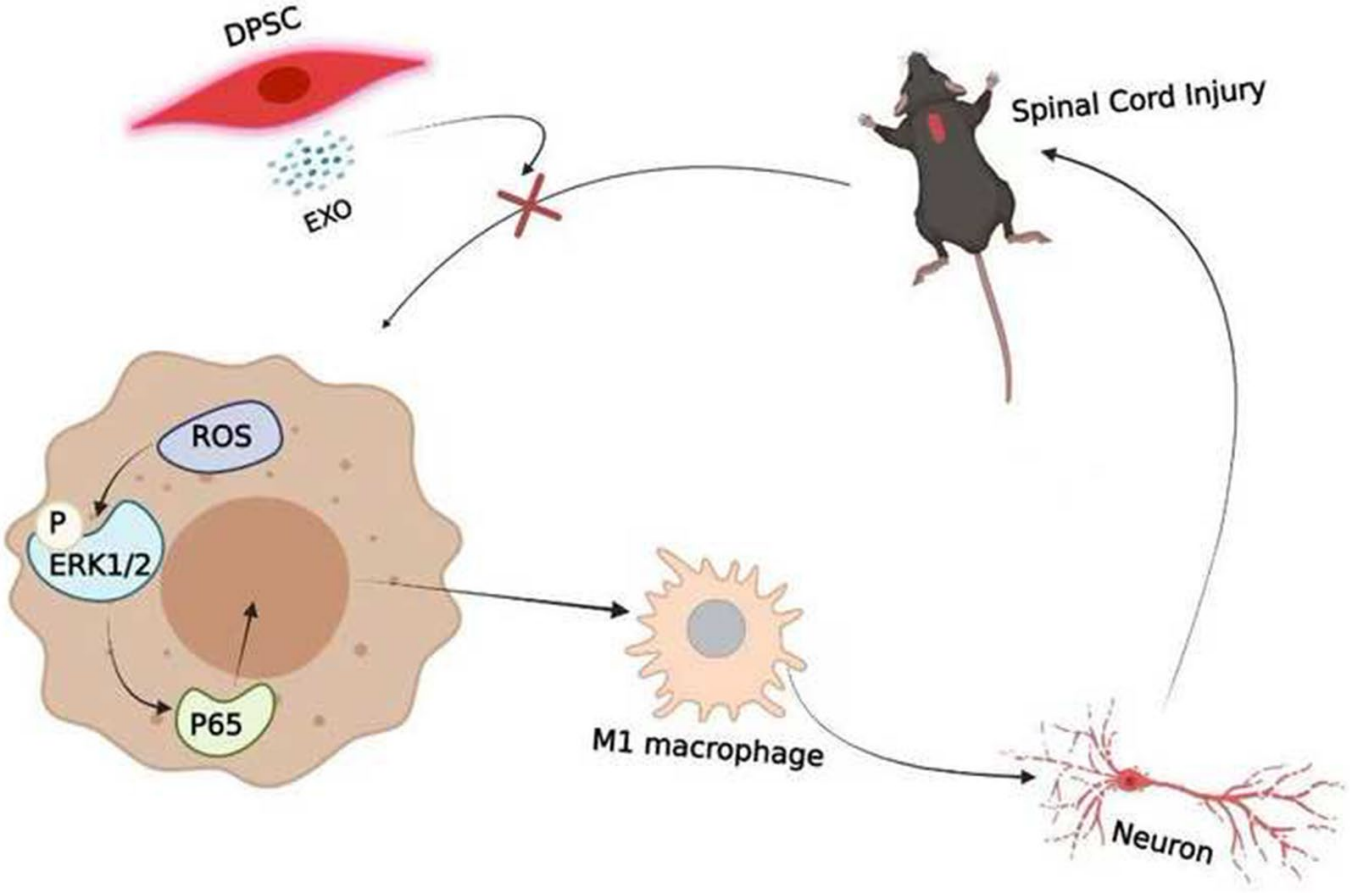

**Supplementary Information:**

The online version contains supplementary material available at 10.1186/s12951-022-01273-4.

## Introduction

Spinal cord injury (SCI) is a complex and devastating clinical condition characterized by deleterious functional loss of sensory and motor functions below the level of the lesion due to neuronal loss and axonal destruction [1–5]. World Health Organization survey data reveal that more than 180,000 patients experience SCI every year [6, 7], and the death rate of acute SCI ranges from 4.4% to 16.7% worldwide. As the aging population of modern society increases, the number of recorded patients with SCI resulting from falls has risen from 16% to 30.5% since 2012 [8–10]. Although several treatment strategies are available, such as surgical operation, pharmacological treatment and hyperbaric oxygenation therapy, no effective therapy is currently available for SCI [5]. Surgical decompression and fixation treatment can restore spine anatomical integrity and stability but has no apparent effect on neuron recovery [11]. Additionally, because of the blood–brain barrier (BBB), the clinical efficiency of therapeutic drugs is also very limited [5, 10, 12, 13]. Therefore, further research on the pathological mechanism and effective treatment measures of SCI needs to be explored.

Tissue damage in SCI results from primary and secondary mechanisms [14]. The primary damage results from immediate primary mechanical injury of axons and neurons [14]. The secondary damage mechanisms include serious neuroinflammation, glutamate excitotoxicity, ischemia, edema, Ca^2+^ overload, compromised energy metabolism and increased reactive oxygen species (ROS) levels [15–18]. Due to increased permeability of the blood–spinal cord barrier, the infiltration of peripheral macrophages to the injury site is increased [12]. Injury triggering inflammation releases ROS, which could exacerbate SCI damage [19]. ROS, including superoxide anions, hydrogen peroxide, peroxynitrite, and hydroxyl radicals, can damage cells and tissues by oxidizing DNA, lipids and proteins, leading to cell dysfunction and cell death and contributing to neurodegeneration [19–21]. Recently, ROS have been described as playing important physiological roles in cellular signaling, such as in the process of cell proliferation, differentiation and metabolism, cancer activity [19, 22–24]. In addition, ROS may also trigger neutrophil-mediated inflammation, which is considered to contribute to secondary damage in SCI [17, 25]. Arnau Hervera performed RNA-seq analysis, and the results showed that ROS could drive the inflammatory and immune response [19]. Therefore, we speculate that there is a cycle between ROS and neuroinflammation after SCI and that this cycle may extend the inflammatory period and inflammatory regions. Disruption of the cycle may be a potential effective treatment by reducing secondary damage. NF-kB and MAPK are the best characterized oxidation–reduction-sensitive signaling pathways, and the increase in ROS production could result in activation of the MAPK and NF-kB pathways [22, 26, 27]. P65 nuclear translocation can be attenuated by inhibiting the activation of the MAPK signaling pathway [28]. Hence, we hypothesized that the ROS-MAPK-NFκB P65 signaling pathway may be an important signaling pathway regulating inflammation.

Recently, stem cell-derived exosomes have gained increasing attention in treating SCI. Exosomes are small particles secreted by living cells and formed from various proteins, including signal proteins, cytoskeletal proteins, and growth factors [29–31]. Exosomes can exercise biological roles through membrane signaling molecular recognition on the membrane surface and membrane fusion (including miRNA and mRNA) for intracellular regulation and extracellular release of active components, thus playing a neuroprotective role, regulating immunity and affecting cell behavior [32–35]. Compared with stem cells, stem cell-derived exosomes not only have most of the biological properties of stem cells but also have the advantage of a small volume and do not easily block microvessels. Exosomes have no growth proliferation capacity and low tumor risk and can penetrate the blood–spinal cord barrier with high membrane transport efficiency. Hence, exosomes have more potential and advantages in the treatment of SCI [10]. Many studies have demonstrated that stem cell-derived exosomes can reduce ROS levels in injured tissue and reduce M1 macrophage polarization in an SCI model [10, 36, 37]. Currently, commonly used exosomes are mainly derived from bone marrow-derived mesenchymal stem cells (BMSCs), umbilical cord mesenchymal stem cells and adipose stem cells. A previous study showed that exosomes derived from dental pulp stem cells (DPSCs) had stronger immunomodulatory effects than exosomes derived from BMSCs [38, 39]. DPSCs are generally extracted from discarded teeth without noninvasiveness, raising no ethical concerns [39]. Similar to the spinal cord, DPSCs also originate from the neural crest [39]. Hence, DPSC-derived exosomes may be an excellent candidate for treating SCI. However, there have been no studies of dental pulp stem cell (DPSC)-derived exosomes in the treatment of SCI. DPSCs can differentiate into a variety of cells in vitro and in vivo, such as neurons and glial cells [40]. It was demonstrated that transplantation of human dental pulp stem cells improves motor nerve function in rat SCI models [40]. DPSCs can also produce neurotrophins to support neural cell survival and promote the homing of endogenous neural stem cells to damaged sites after transplantation [41]. DPSCs have low immunogenicity and strong immunoregulatory ability [41]. However, the ability of DPSC-Exos to promote motor nerve function recovery in SCI models is unclear. Many studies suggest that M1 macrophages can secrete inflammatory cytokines, such as IL-1β and TNF-α, which further damage the cells [42–44]. Hence, we hypothesized that DPSC-derived exosomes can inhibit M1 macrophage polarization through the ROS-MAPK-NFκB P65 signaling pathway. The purpose of the present study was to investigate the neuroprotective mechanism of DPSC-Exos in treating SCI and to further explore whether DPSC-Exos could inhibit M1 macrophage polarization through the ROS-MAPK-NFκB P65 signaling pathway.

## Methods and materials

### Preparation of DPSCs

Impacted human wisdom teeth were collected at the Dental Clinic of Beijing Stomatological Hospital under approved guidelines (BSH [2015] D-15). The patients’ ages ranged from 19 to 29 years old. All patients provided written informed consent to participate. After cleaning tooth surfaces, the pulp chambers were exposed by cutting around the cementoenamel junction using a sterilized dental fissure burst.

DPSCs were isolated as described in previous work [39, 45]. Dental pulp was collected from the crown and root. After being cut into 1-mm pieces, the pulp was transferred into collagenase and dispase (Sigma–Aldrich, St. Louis, MO) and digested for 40 min at 37 °C in humid air with 5% CO_2_. After washing with PBS, the pulp pieces were transferred into a cell culture flask to culture with alpha-modified Eagle’s medium (alpha-MEM; Gibco; Thermo Fisher Scientific, Grand Island, NY) containing 15% fetal bovine serum (FBS; Thermo Fisher Scientific) at 37 °C in humid air with 5% CO_2_. After 14 days of culture, the cells were harvested, and the surface molecule expression profiles and multilineage differentiation of MSCs were determined. (data shown in Additional file 1: Fig. S1).

### DPSCs-Exo isolation and characterization

A total of 5 × 10^6^ passage 4 DPSCs were seeded in a T-150 flask and cultured until 80% confluence. After washing the cells twice with PBS, α-MEM without FBS was added to the flask, and the cells were cultured for 48 h. Supernatants were collected. Exosomes were isolated from the supernatants of passage 4 DPSCs by differential centrifugation (300 ×*g* for 10 min; 2000 ×*g* for 10 min; 10,000 ×*g* for 30 min, 100,000 ×*g* for 70 min) and washing of the exosomes with PBS (100,000 ×*g* for 70 min). All centrifugations were performed at 4 °C. Subsequently, the exosomes were resuspended in PBS and stored at − 80 °C.

Exosome morphology was observed with transmission electron microscopy. Ten microliters of the exosome suspension was loaded onto formvar/carbon-coated grids at 25 °C for 10 min. Then, excess fluid was removed, exosomes were negatively stained with 3% aqueous phosphotungstic acid for 5 min, and the exosome-containing grids were observed using a transmission electron microscope (HT7700, HITACHI, Tokyo, Japan) operating at 80 kV.The expression of exosome markers was also detected by western blot. Analysis of size distribution was performed using a Nanoparticle Tracker Analyzer (NTA, ZetaView, Particle Metrix Inc., Germany).

### RAW264.7 cell culture and experimental design

RAW264.7 cell lines were used in this study. A total of 1 × 10^5^ cells were seeded in the wells of 6-well plates and cultured in basic DMEM containing 10% FBS (Sigma–Aldrich, St. Louis, MO). The cells cultured in the above medium were defined as M0 macrophages.

H_2_O_2_ was used to treat RAW264.7 cells to investigate whether ROS signaling supports macrophage polarization and to determine the role of ROS concentration in the macrophage polarization process. RAW264.7 cells were randomly divided into seven groups: the control, 50 μM, 100 μM, 150 μM, 200 μM, 250 μM and 500 μM groups. The concentration of H_2_O_2_ we used in the following study was 500 μM.

Lipopolysaccharide (LPS, 100 ng/mL, Sigma–Aldrich) was used to treat RAW264.7 cells. RAW264.7 cells were randomly divided into three groups: the control, LPS and LPS + Exos groups. The LPS + Exos group was treated with both LPS and DPSC-Exos. DPSC-Exos were used to grow RAW264.7 cells at a concentration of 10 μg/ml.

A 10 μmol/ml nonselective inhibitor of the ROS-producing flavoenzyme diphenyleneiodonium (DPI) (Selleckchem, Houston, TX, USA) was used to inhibit ROS production. RAW264.7 cells were randomly divided into four groups: the control, LPS, LPS + DPI, and LPS + DPI + H_2_O_2_ groups. Furthermore, RAW264.7 cells were also randomly divided into three groups: the control, H_2_O_2_ and H_2_O_2_ + DPI groups.

To dissect the role of P65, the antagonist BAY 11–7082 (Beyotime Shanghai, China) (10 μmol/ml) was used to suppress the P65 signaling pathway. RAW264.7 cells were randomly divided into three groups: the control, H_2_O_2_ and H_2_O_2_ + BAY 11–7082 groups. RAW264.7 cells were also randomly divided into three groups: the control, LPS and LPS + BAY 11–7082 groups.

To dissect the role of MAPK, PD98059 (AbMole Bioscience Inc, Houston, TX, USA) (10 μmol/ml) was used to suppress the ERK1/2 signaling pathway. RAW264.7 cells were randomly divided into three groups: the control, H_2_O_2_ and H_2_O_2_ + PD98059 groups. Furthermore, RAW264.7 cells were also randomly divided into three groups: the control, LPS and LPS + PD98059 groups.

### RNA extraction and quantitative RT–PCR analysis

After 2, 4, and 6 h of culture with LPS or H_2_O_2_ in vitro, total RNA was extracted from RAW264.7 cells using TRIzol reagent (Invitrogen, USA) according to the manufacturer's protocol. RNA concentration and sample purity were measured using an ultraviolet spectrophotometer (UVS-99, ACTGene, USA). The purity of extracted RNA was approximately 2.0. Complementary DNA (cDNA) was synthesized from total RNA using StarScript II First-strand cDNA Synthesis Mix with gDNA Remover (GenStar, China). The expression level of the gene was detected by qRT–PCR using RealStar Green Fast Mixture with ROX II (GenStar, China).

The ACTIN level was used to normalize the gene-specific expression levels. PCRs in 20 µl were carried out in triplicate. The PCR conditions were as follows: initial denaturation at 95 °C for 2 min followed by 40 cycles, each consisting of 15 s at 95 °C, 30 s at 60 °C, 30 s at 72 °C and then 1 cycle for the melting curve. The primer sequences were as follows: m-Actin, forward, 5′-CCTCACTGTCCACCTTCC-3′ and reverse 5′- GGGTGTAAAACGCAGCTC-3′; m-iNOS, forward 5′-GCCCAGGAGGAGAGAGAT-3′ and reverse 5′-GCAAAGAGGACTGTGGCT-3′.

### Flow cytometry

After 2, 4, 6, and 24 h of LPS or LPS + Exos pretreatment and removal of the supernatant, the cells were washed twice with PBS. Then, after incubation with H2DCFDA (C6827, Thermo Fisher Scientific, Grand Island, NY) for 60 min at 37 °C in the dark, the cells were collected and washed twice with PBS and resuspended in 500 μL of PBS. ROS was detected by flow cytometry (FACS Calibur, BD, USA).

After 24 h of culture with LPS, LPS + Exos, H_2_O_2_, LPS + DPI, H_2_O_2_ + DPI, LPS + P65 inhibitor, H_2_O_2_ + P65 inhibitor, LPS + PD98059 or H_2_O_2_ + PD98059, the expression levels of M1 macrophage surface markers were detected by flow cytometry. At 24 h post-treatment, cells were harvested and washed twice with PBS and then incubated with anti-CD86-PE (Thermo Fisher 12-0862-82) according to the manufacturer’s protocol. After incubation with anti-CD86-PE for 30 min at 4 °C in the dark, the cells were washed twice with PBS and resuspended in 500 μL of PBS. Cell surface expression was detected using flow cytometry.

After 24 h of culture with LPS, LPS + Exos, H_2_O_2_, LPS + DPI, H_2_O_2_ + DPI, LPS + PD98059 or H_2_O_2_ + PD98059, P65 was detected by flow cytometry. At 24 h post-treatment, cells were harvested and washed twice with PBS and then fixed for 15 min at room temperature with 4% formaldehyde. Cells were washed twice with 1 × PBS. Cells were permeabilized by adding ice-cold 100% methanol slowly to prechilled cells while gently vortexing to a final concentration of 90% methanol, followed by permeabilization for a minimum of 10 min on ice. The cells were washed by centrifugation in excess 1 × PBS to remove the methanol. The cells were incubated with anti-p65-Alexa Fluor 488 (CST 494455) for 1 h at room temperature in the dark. Cells were washed twice by centrifugation in antibody dilution buffer and resuspended in 500 µL of PBS. P65 was then detected using flow cytometry.

At 3 days and 5 days after SCI, 3-mm spinal cord segments were resected from mice centered on the injury epicenter. After washing with PBS, the endorachis and blood vessels were removed from the spinal cord tissues and placed under a microscope. After being cut into small pieces, the spinal cord tissues were transferred into culture medium with 2% papain for 25 min at 37 °C and vertexed once every five minutes. After centrifugation at 200 rpm for two min, the supernatant was collected. After centrifugation at 1000 rpm for five min,the supernatant was discarded. Cells were washed twice with 1 × PBS. Then, the cells were incubated with H2DCFDA (C6827, Thermo Fisher Scientific, Grand Island, NY) for 60 min at 37 °C in the dark. The cells were collected and washed twice with PBS and resuspended in 500 μL of PBS. ROS was detected by flow cytometry (FACS Calibur, BD, USA).

### Western blot

Exosomes were lysed in RIPA buffer with protease inhibitors. Cells and spinal cord tissues were lysed in RIPA buffer with protease and phosphatase inhibitors (Beyotime Shanghai, China). The concentrations of proteins were measured using a Pierce™ BCA Protein Assay Kit (Rockford, IL, USA). Then, the preparations were mixed with 5 × SDS loading buffer, denatured at 100 °C for 10 min and loaded onto a 10% SDS–PAGE gel for electrophoresis. After electrophoresis and washing with TBS-T, proteins were transferred to 0.22-μm polyvinylidene difluoride membranes (Bio–Rad, USA) and then blocked with 5% nonfat milk. The membranes were incubated with the following primary antibodies overnight at 4 °C: CD9 antibody (diluted 1:1000, Abcam, catalog number: ab92726), CD63 antibody (diluted 1:1000, Abcam, catalog number: ab134045), tubulin (diluted 1:3000, Servicebio, catalog number: GB11017B), albumin (diluted 1:1000, Abcam, catalog number: ab151742), CD73 antibody (diluted 1:1000, Abcam, catalog number: ab133582), p42/44 antibody (diluted 1:1000, CST, catalog number: 9102), and p-p42/44 antibody (diluted 1:1000, CST, catalog number: 9101). The next day, the membranes were washed with TBST four times for 5 min each time, and then the membranes were incubated for 1 h with secondary antibody (1:6000) at room temperature. After washing the membranes with TBST more than four times, the blots were detected using electrochemiluminescence plus reagent (Invitrogen, USA). Protein band densities were assessed by CS Analyzer software (Version 3.00.1011, ATTO & Rise Corporation).

### Mice

Six- to eight-week-old female C57BL/6 mice were obtained from Charles River Laboratories (Beijing, China). All mice weighed 17.4–22 g at the time of surgery. All animals were maintained under constant temperature and humidity conditions and experienced a 12-h/12-h light/dark cycle. All animal experiments were performed under protocols approved by the Institutional Animal Care and Use Committee of the Beijing Institute of Radiation Medicine (IACUC-DWZX-2020-717).

### Animal model of spinal cord injury

A contusive SCI model was established using a New York University Impactor, as previously described [46]. Briefly, mice were anesthetized with an intraperitoneal injection of pentobarbital (50 mg/kg). A laminectomy was performed at the T11–12 level. After laminectomy and exposing the dorsal surface of the cord, a 10-g rod was dropped from a height of 6.25 mm, hitting the stabilized spine and damaging the cord. After cord injury was achieved, the incision was sutured layer by layer.

### Treatment of hDPSC-derived exosomes in SCI

All SCI mice were randomly assigned to two groups, and each group included 12 mice. Thirty minutes after the surgery, 100 μl of PBS or 200 μg/100 μl hDPSC-derived exosomes were administered through the tail vein. Mice were placed in a controlled environment. Until reflex bladder emptying ability was restored, manual bladder emptying was performed twice each day.

### Spinal cord function evaluation

At 1, 3, 5, 7, 14, 21 and 28 days post-SCI, the recovery of spinal cord function was evaluated through Basso Mouse Scale (BMS) scoring. The BMS score ranges from 0 to 9 points; 0 points represents full paralysis, and 9 points represents complete normality [47]. The mice were kept on a flat area and allowed free movement for 4 min. Hindlimb movement function was assessed by two independent observers. For each mouse, the mean BMS score was adopted as the individual BMS score.

### Immunofluorescent staining

#### Cell immunofluorescent staining

The cultured cells were washed with PBS three times. After fixation with 4% polyformaldehyde for 15 min, the cells were washed with PBS three times. Next, the cells were permeabilized for 20 min with 0.5% Triton- × 100 and then washed with PBS three times. The cells were blocked with 5% BSA for 30 min and then incubated with anti-p65 antibody in a humidified chamber overnight at 4 °C. Cells were washed with PBS three times and then incubated with secondary antibody for 60 min at room temperature in the dark. After washing three times, the cells were incubated with DAPI solution in the dark for 5 min to stain the cell nuclei. Samples were photographed with an OLYMPUS CKX53 (Tokyo Japan).

#### Tissue immunofluorescent staining

A 10-mm spinal cord segment was resected from mice at 3, 5 and 28 days after surgery. The spinal cord tissues were soaked in 4% paraformaldehyde for 24 h and then transferred into 30% sucrose solution until the tissue sank. Frozen spinal cord tissues were cut into 10-μm slices (Leica, Germany). Slices were incubated with anti-CD86 antibody, anti-P65 antibody, anti-NF200 antibody, and anti-NEUN antibody in a humidified chamber overnight at 4 °C, incubated with the secondary antibody marked with HRP, and then incubated with CY3-TSA or FITC-TSA solution. An inverted fluorescence microscope (Leica DM6000, Wetzlar, Germany) was applied for photographing samples and further analysis.

### Statistical analysis

Data analysis was performed using GraphPad Prism 7.0 (San Diego, CA, USA). The data are presented as the mean ± SD. Two-way ANOVA was adopted to compare BMS scores between groups over time. The remaining data were analyzed using Student’s t test or one-way ANOVA with Tukey’s multiple comparison test. A P value less than 0.05 was considered to be significant in all analyses.

## Results

### ROS have a concentration-dependent effect on M1 macrophage polarization, and LPS can promote M1 macrophage polarization by inducing ROS production

**Fig. 1:**
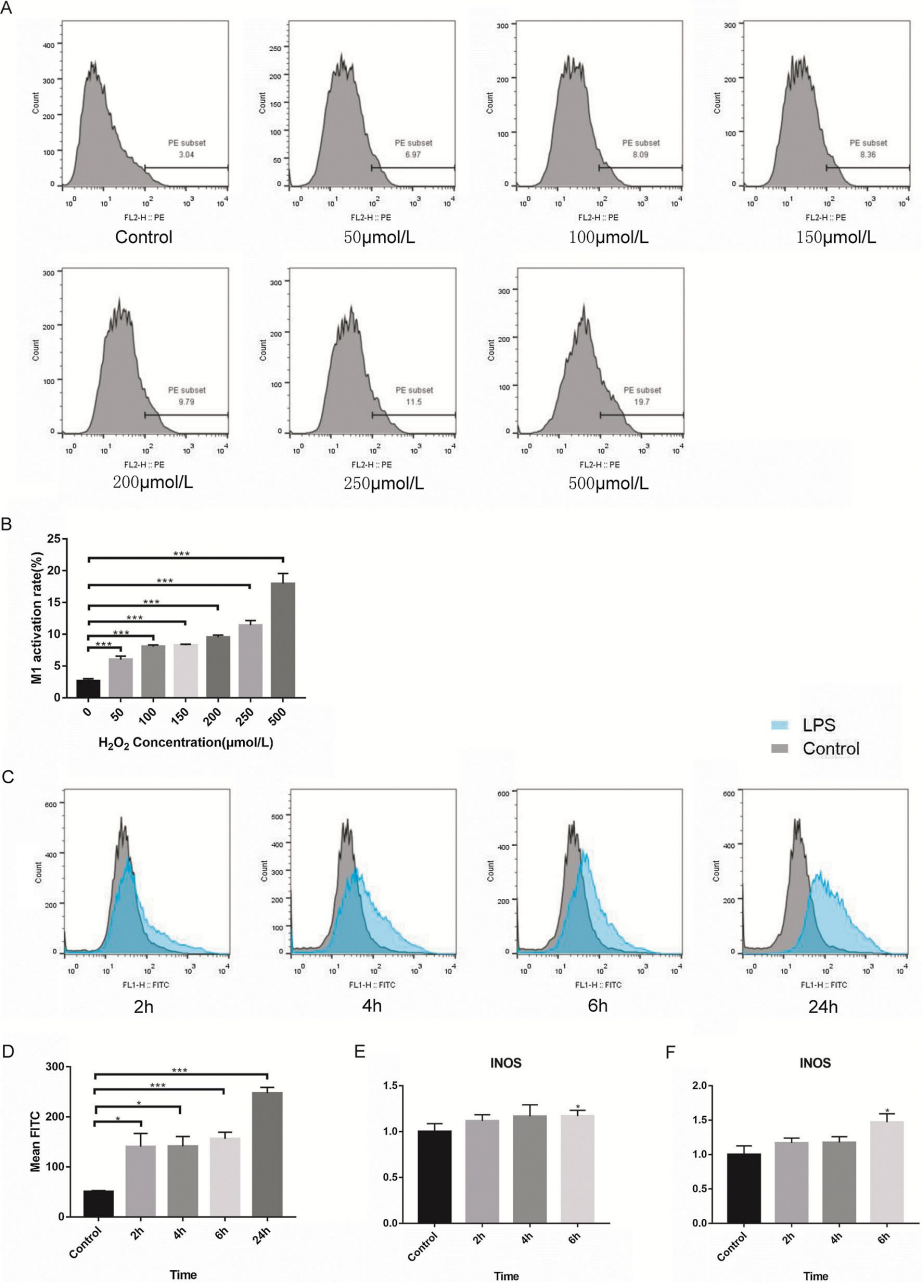
ROS has a concentration-dependent effect on macrophages M1 polarization, and LPS can promote macrophages M1 polarization by inducing ROS production. **A** and **B** Raw264.7 cells cultured with different concentration of H_2_O_2_ (ranging from 50 μmol/L to 500 μmol/L) for 24 h. M1 polarization rate was increased with an increased H_2_O_2_ concentration in the culture medium. **C** and **D** Raw264.7 cells cultured with LPS for 2, 4, 6 and 24 h. ROS level was detected by using FACS with a flow cytometry. **E** and **F** Raw264.7 cells cultured with LPS or H_2_O_2_ for 2, 4 and 6 h.The mRNA (iNOS) relative expression level of M1 phase is detected. Both LPS and H_2_O_2_ markedly upregulated the expression of iNOS at 6 h after LPS and H_2_O_2_ treatment. *p < 0.05, ***p < 0.01

In vitro, we cultured RAW264.7 cells with different concentrations of H_2_O_2_ (ranging from 50 μmol/L to 500 μmol/L) for 24 h. The flow cytometry analysis results revealed that there was a concentration-dependent promoting effect on M1 macrophage polarization. The M1 polarization rate was increased with an increased H_2_O_2_ concentration in the culture medium (0 μmol/L:2.647 ± 0.358, 50 μmol/L:6.003 ± 0.5424, 100 μmol/L:8.05 ± 0.2787, 150 μmol/L:8.263 ± 0.1656, 200 μmol/L:9.56 ± 0.3236, 250 μmol/L:11.4 ± 0.755, 500 μmol/L:17.97 ± 1.617) (Fig. 1A, B). We also detected the effect of a higher H_2_O_2_ concentration (1 mmol/L) on M1 polarization and found that it reached a toxic level and mainly induced cell death (data shown in Additional file 2: Fig. S2). Hence, ROS can induce M1 macrophage polarization within a certain range of concentrations and have a concentration-dependent effect.

Then, we cultured RAW264.7 cells with LPS for 2, 4, 6 and 24 h. ROS levels were detected using FACS, and the results showed that ROS levels were significantly increased as early as 2 h after LPS treatment (0 h: 50.5 ± 2.052, 2 h:140.7 ± 26.31, 4 h: 141.3 ± 19.43, 6 h: 156.3 ± 13.05, 24 h: 247.7 ± 11.5) (Fig. 1C, D). Moreover, total RNA was extracted from RAW264.7 cells, and quantitative RT–PCR analysis was performed. We found that both LPS (0 h vs 6 h:1 ± 0.086 vs 1.17 ± 0.061) and H_2_O_2_ (0 h vs 6 h:1 ± 0.126 vs 1.47 ± 0.122)markedly upregulated the expression of iNOS, a marker of M1 macrophages, at 6 h after LPS and H_2_O_2_ treatment (Fig. 1E, F). These results suggest that ROS may mediate LPS-induced M1 macrophage polarization.

### ROS activate M1 macrophage polarization through the MAPK-NFκB P65 signaling pathway

**Fig. 2:**
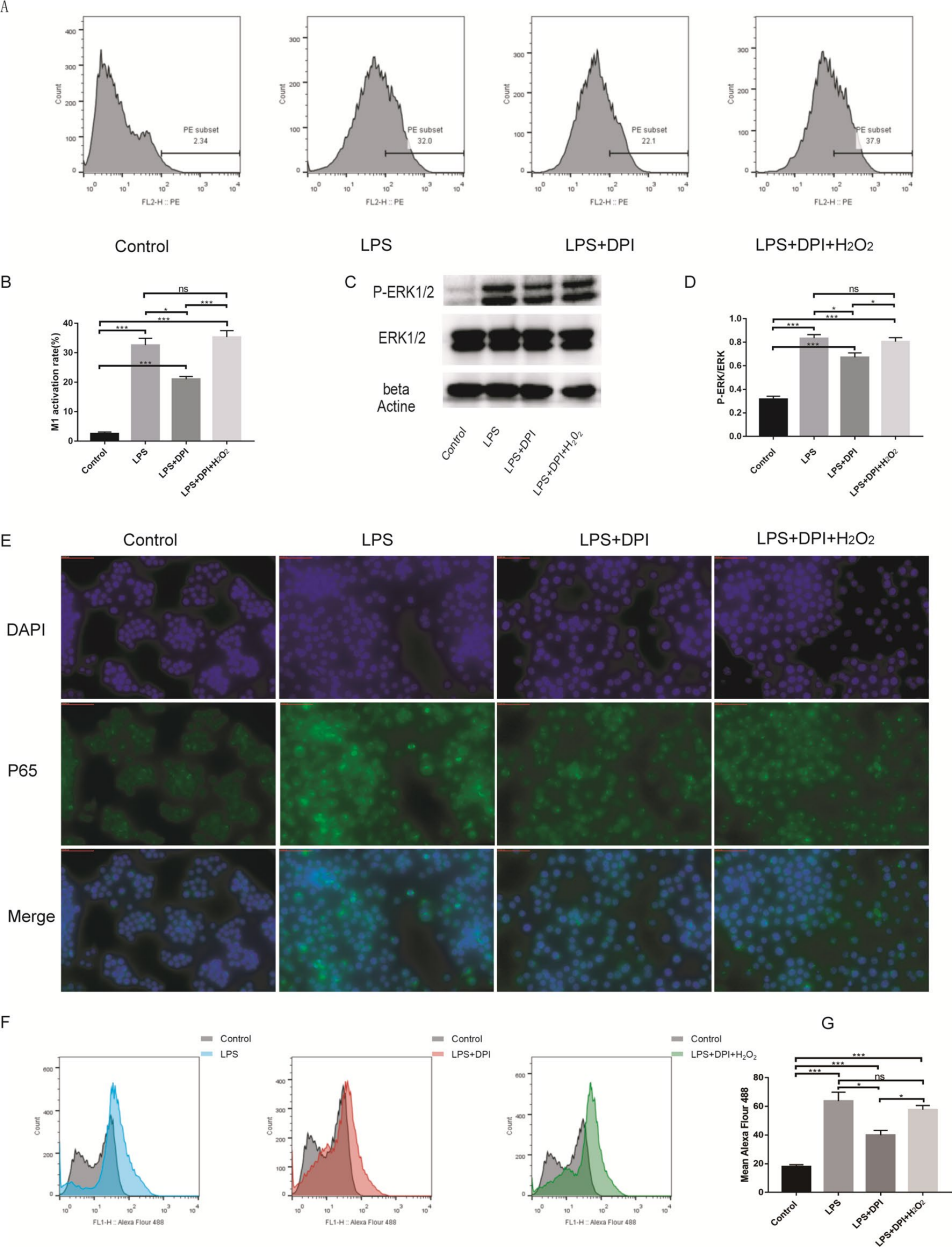
DPI can suppress macrophages M1 polarization and MAPK-NFκB P65 signaling pathway. **A** and **B** Raw264.7 cells were treated with LPS, LPS + DPI or LPS + DPI + H_2_O_2_. M1 macrophages surface expression (CD86) was detected using using flow cytometry. The LPS + DPI group had a markedly lower M1 polarization rate than the LPS group. Furthermore, the LPS + DPI + H_2_O_2_ group had a significantly increased M1 polarization rate compared with the LPS + DPI group. **C** and **D** The ERK1/2 and p-ERK1/2 protein expression in Raw264.7 cells was either investigated. LPS upregulated the P-ERK/ERK level in RAW264.7 cells and that DPI downregulated the LPS-induced increase in the P-ERK/ERK level. The LPS + DPI + H2O2 group had a higher P-ERK/ERK level than the LPS + DPI group. **E–G** P65 protein expression are determined by immunofluorescences and flow cytometry. LPS could increase P65 expression. DPI had a marked inhibitory effect on the increase in P65 fluorescence, and H2O2 counteracted the inhibitory effect of DPI.*p < 0.05, ***p < 0.01

**Fig. 3:**
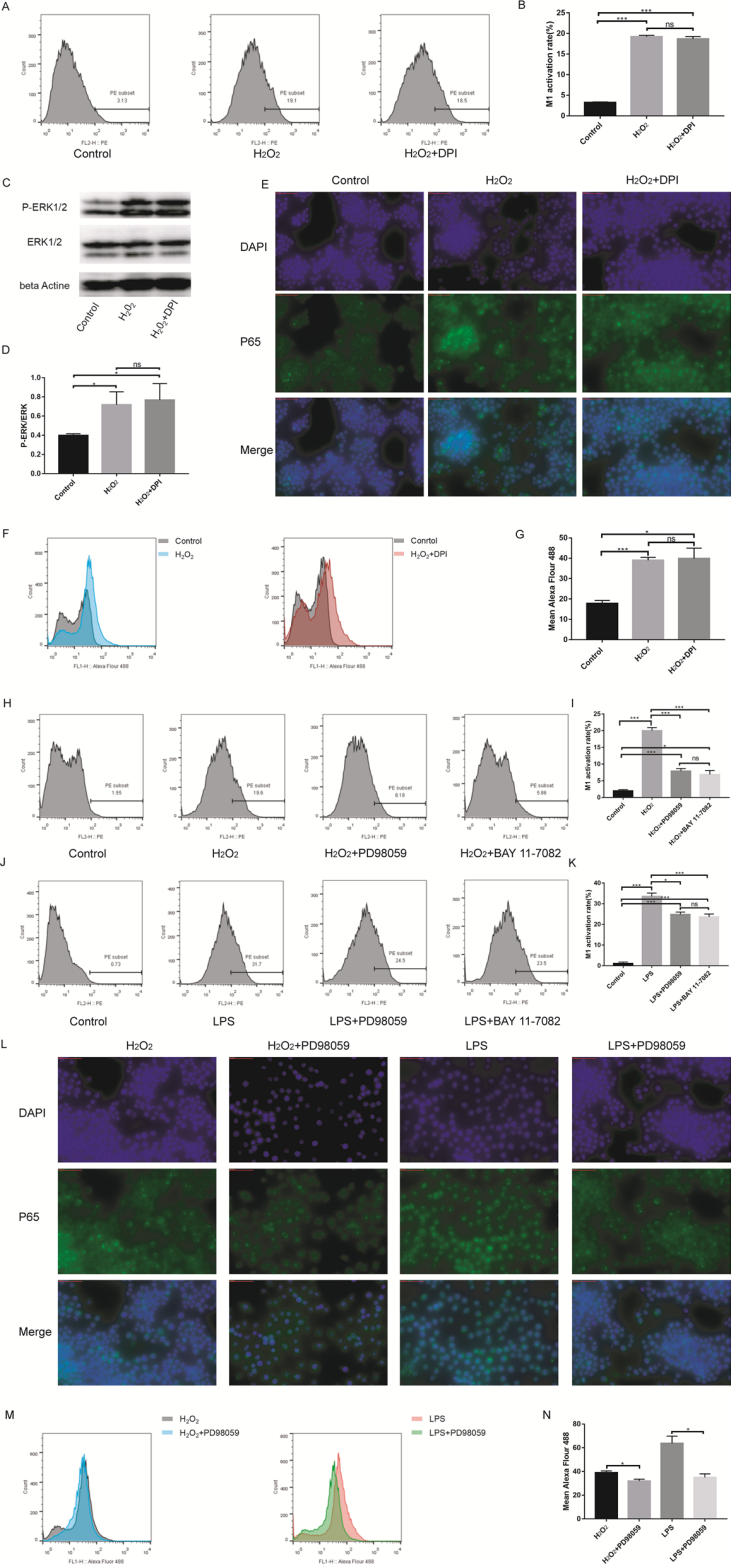
ROS activates macrophages M1 polarization through MAPK-NFκB P65 signaling pathway. **A** and **B** Raw264.7 cells were treated with H_2_O_2_ or H_2_O_2_ + DPI. M1 macrophages surface expression (CD86) was detected using flow cytometry. H2O2 could promote M1 macrophage polarization, but DPI had no effect on M1 macrophage polarization induced by H2O2. **C** and **D** The ERK1/2 and p-ERK1/2 protein expression in Raw264.7 cells was either investigated. H2O2 could increase the P-ERK/ERK level and that DPI had no influence on the phosphorylation of ERK in cells cultured with H2O2. **E–G** P65 protein expression are determined by immunofluorescences and flow cytometry. P65 expression was also increased after H2O2 treatment for 24 h, and DPI could not suppress the P65 fluorescence increase caused by H2O2. **H** and **I** Raw264.7 cells were treated with H_2_O_2_, H_2_O_2_ + BAY 11–7082 or H_2_O_2_ + PD98059. **J** and **K** Raw264.7 cells were treated with LPS, LPS + BAY 11–7082 or LPS + PD98059. **L–N** P65 protein expression are determined by immunofluorescences and flow cytometry. M1 macrophages surface expression (CD86) was detected using using flow cytometry. macrophage M1 polarization caused by both LPS and H2O2 could be suppressed by the P65 inhibitor. PD98059, an inhibitor of MEK, could also inhibit P65 expression and that M1 macrophage polarization was increased by LPS or H2O2. *p < 0.05, ***p < 0.01

To verify that ROS may mediate LPS-induced M1 macrophage polarization, we inhibited the production of ROS induced by LPS. Diphenyleneiodonium (DPI) is a nonselective inhibitor of ROS-producing flavoenzymes. The LPS + DPI group had a markedly lower M1 polarization rate than the LPS group (LPS + DPI vs LPS: 21.03 ± 0.945 vs 32.6 ± 2.358). Furthermore, the LPS + DPI + H_2_O_2_ group had a significantly increased M1 polarization rate compared with the LPS + DPI group (LPS + DPI + H_2_O_2_ vs LPS + DPI:35.33 ± 2.228 vs 21.03 ± 0.945) (Fig. 2A, B). Western blot results showed that LPS upregulated the P-ERK/ERK level in RAW264.7 cells and that DPI downregulated the LPS-induced increase in the P-ERK/ERK level (LPS + DPI vs LPS: 0.671 ± 0.081 vs 0.833 ± 0.031). The LPS + DPI + H_2_O_2_ group had a higher P-ERK/ERK level than the LPS + DPI group (LPS + DPI + H_2_O_2_ vs LPS + DPI:0.804 ± 0.033 vs 0.671 ± 0.081) (Fig. 2C, D). Flourescence microscopy and flow cytometry were used to detect the expression of P65, and the results showed that LPS could increase P65 expression (Control vs LPS: 17.87 ± 1.419 vs 63.73 ± 6.152). DPI had a marked inhibitory effect on the increase in P65 fluorescence (LPS vs LPS + DPI: 63.73 ± 6.152 vs 39.87 ± 3.371), and H_2_O_2_ counteracted the inhibitory effect of DPI (LPS + DPI + H_2_O_2_ vs LPS + DPI: 57.7 ± 2.858 vs 39.87 ± 3.371) (Fig. 2E–G). Furthermore, we demonstrated that H_2_O_2_ could promote M1 macrophage polarization (Control vs H_2_O_2_: 3.227 ± 0.158 vs 19.2 ± 0.361), but DPI had no effect on M1 macrophage polarization induced by H_2_O_2_ (H2O2 + DPI vs H_2_O_2_: 18.67 ± 0.588 vs 19.2 ± 0.361) (Fig. 3A, B)_._ Western blot results also showed that H_2_O_2_ could increase the P-ERK/ERK level (Control vs H2O2: 0.397 ± 0.018 vs 0.718 ± 0.134) and that DPI had no influence on the phosphorylation of ERK in cells cultured with H_2_O_2_(H2O2 + DPI vs H2O2: 0.767 ± 0.171 vs 0.718 ± 0.134) (Fig. 3C, D). P65 expression was also increased after H_2_O_2_ treatment for 24 h (Control vs H2O2: 17.87 ± 1.419 vs 39 ± 1.473), and DPI could not suppress the P65 fluorescence increase caused by H_2_O_2_ (H2O2 + DPI vs H2O2: 39.83 ± 5.16 vs 39 ± 1.473) (Fig. 3E–G).

P65 was an important factor in M1 macrophage polarization caused by both LPS and H_2_O_2_. To further support our findings, the antagonist BAY 11–7082 (a P65 inhibitor) was introduced. The flow cytometry analysis results revealed that macrophage M1 polarization caused by both LPS and H_2_O_2_ could be suppressed by the P65 inhibitor (LPS + BAY11-7082 vs LPS: 23.6 ± 1.453 vs 33.5 ± 1.67; H_2_O_2_ + BAY11-7082 vs H_2_O_2_: 6.89 ± 1.195 vs 20 ± 0.94) (Fig. 3H–K). The results also revealed that PD98059, an inhibitor of MEK, could also inhibit P65 expression (LPS + PD98059 vs LPS: 24.77 ± 1.222 vs 33.5 ± 1.67; H_2_O_2_ + PD98059 vs H_2_O_2_: 7.903 ± 0.773 vs 20 ± 0.94) and that M1 macrophage polarization was increased by LPS or H_2_O_2_ (LPS + PD98059 vs LPS: 35 ± 3.032 vs 63.73 ± 6.152; H_2_O_2_ + PD98059 vs H_2_O_2_: 31.93 ± 1.626 vs 39 ± 1.473) (Fig. 3H–N).

### Isolation and identification of DPSC-derived exosomes

**Fig. 4:**
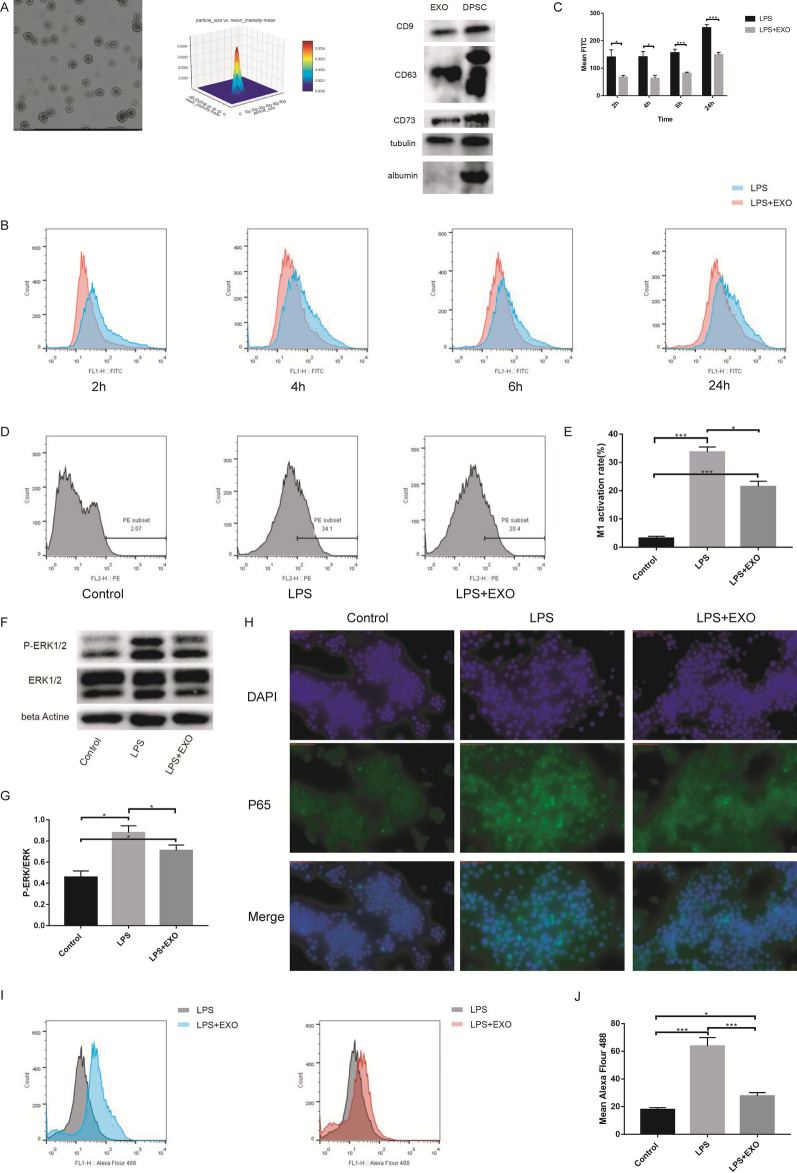
Characteristics of DPSCs derived Exosomes. DPSC derived exosomes could inhibit macrophages M1 polarization through MAPK-NFκB P65 signaling pathway in vivo. **A** Representative images of exosomes are observed under transmission electron microscopy (TEM). Size distribution of extracellular vesicle is measured by nanoparticle tracking analysis (NTA, ZetaView, Particle Metrix Inc., German). Western-blotting analysis of indicated proteins is detected, including CD63, CD9, tubulin, albumin and the MSC marker CD73. **B** and **C** Raw264.7 cells were treated with LPS or LPS + EXO for 2, 4, 6 and 24 h. ROS level was detected by using FACS with a flow cytometry. The LPS + Exos group had a lower ROS level than the LPS group. **D** and **E** Raw264.7 cells were treated with LPS or LPS + EXO for 24 h. M1 macrophages surface expression (CD86) was detected using using flow cytometry. DPSC-derived exosomes could lower the LPS-induced increase in the M1 polarization rate. **F** and **G** The ERK1/2 and p-ERK1/2 protein expression in Raw264.7 cells was either investigated. LPS + Exos group had a lower P-ERK/ERK level than the LPS group. **H–J** P65 protein expression are determined by immunofluorescences and flow cytometry. DPSC-derived exosomes also inhibited the increase in P65 fluorescence induced by LPS. *p < 0.05, ***p < 0.01

Exosomes were isolated with differential centrifugation from passage 4 DPSC culture supernatant. The structure of exosomes was photographed with TEM (Fig. 4A), which showed numerous saucer-shaped vesicles. TRPS analysis showed a diameter of approximately 100 nm (Fig. 4A) and a concentration of 2.2E + 11 particles/ml. The expression of the exosome markers CD63 and CD9, tubulin, albumin and the MSC marker CD73 in the DPSC-Exo samples (Fig. 4A) and DPSC proteins (Fig. 4A) was verified by western blot. These results demonstrated that the isolated extracellular vesicles were hDPSC-derived exosomes. Furthermore, the protein concentration was 2.0 μg/μl in the exosomes quantified using the BCA protein assay kit.

### DPSC-derived exosomes could inhibit M1 macrophage polarization through the MAPK-NFκB P65 signaling pathway

Additionally, we used exosomes to treat RAW264.7 cells to observe the protective effect on LPS-induced cell damage in vitro. The LPS + Exos group had a lower ROS level than the LPS group at 2 h (LPS vs LPS + EXO: 140.667 ± 26.312 vs 66.7 ± 6.482), 4 h (LPS vs LPS + EXO: 141.333 ± 19.425 vs 63.267 ± 11.188), 6 h (LPS vs LPS + EXO: 156.333 ± 13.051 vs 81.133 ± 4.409) and 24 h (LPS vs LPS + EXO: 247.667 ± 11.054 vs 149.333 ± 8.505) after treatment (Fig. 4B, C). The flow cytometry analysis results showed that DPSC-derived exosomes could lower the LPS-induced increase in the M1 polarization rate of RAW264.7 cells (LPS vs LPS + EXO: 33.67 ± 1.79 vs 21.43 ± 1.922) (Fig. 4D, E). Western blot results revealed that the LPS + Exos group had a lower P-ERK/ERK level than the LPS group(LPS vs LPS + EXO: 0.877 ± 0.064 vs 0.709 ± 0.051) (Fig. 4F, G). DPSC-derived exosomes also inhibited the increase in P65 fluorescence induced by LPS (LPS vs LPS + EXO: 63.73 ± 6.152 vs 27.6 ± 2.506) (Fig. 4H–J). Hence, all the above mentioned results demonstrated that DPSC-derived exosomes could inhibit M1 macrophage polarization through the ROS-MAPK-NFκB P65 signaling pathway in vitro.

### DPSC-derived exosomes can inhibit the ROS-MAPK-NFκB P65 signaling pathway in vivo

**Fig. 5:**
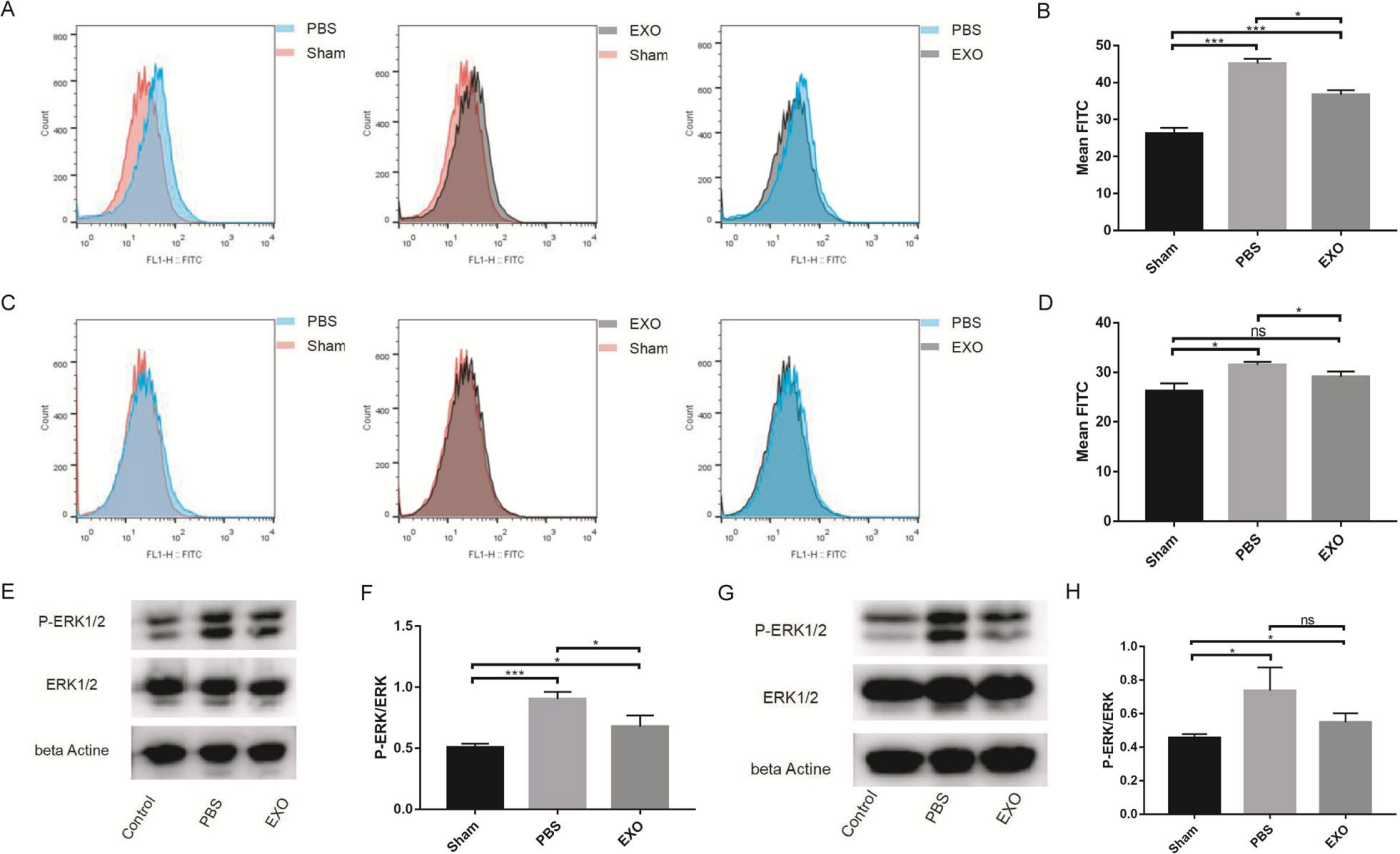
DPSC derived exosomes can inhibit ROS-MAPK-NFκB P65 signaling pathway in vivo. ROS level was detected by using FACS with a flow cytometry. **A** and **B** Mice are analyzed at 3 days after injured spinal cord. **C** and **D** Mice are analyzed at 5 days after injured spinal cord. There was a marked increase in ROS levels in the injured spinal cord at 3 days and 5 days. **E** and **F** At 3 days, the ERK1/2 and p-ERK1/2 protein expression in spinal tissues was investigated. **G** and **H** At 5 days, the ERK1/2 and p-ERK1/2 protein expression in spinal tissues was either investigated. There was also a higher P-ERK/ERK level in the SCI group than in the sham group at 3 days and 5 days. *p < 0.05, ***p < 0.01.

**Fig. 6:**
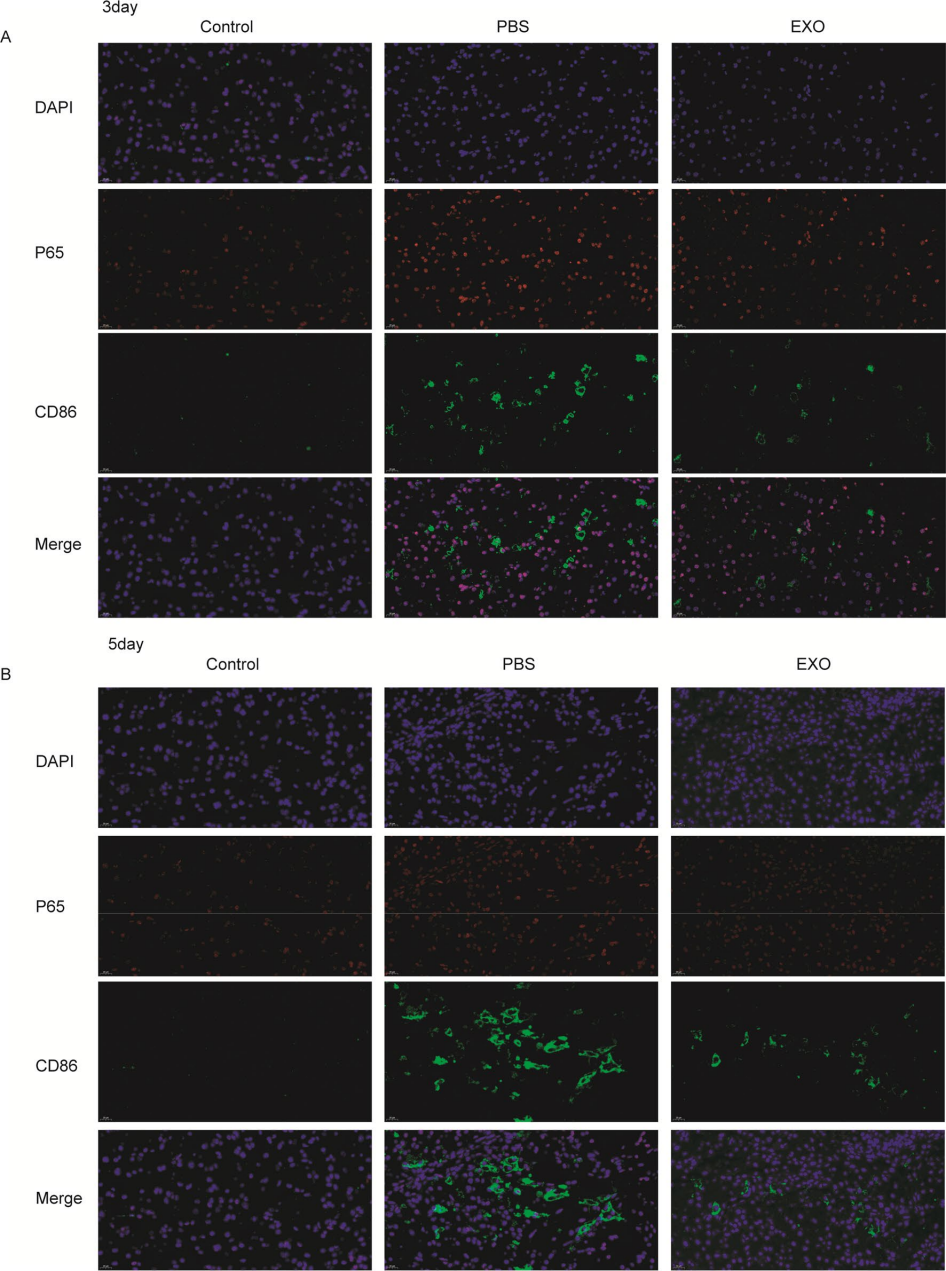
DPSC derived exosomes can inhibit macrophages M1 polarization in mice. **A** At 3 days after injured spinal cord, P65 and CD86 were determined by immunofuorescence in different groups. **B** At 5 days after injured spinal cord, P65 and CD86 were determined by immunofluorescences in different groups. P65 and CD86 fluorescence intensity around the injury site was increased in the SCI group 3 days and 5 days after SCI

At 3 and 5 days after spinal cord injury, flow cytometry analysis was performed. The results showed that there was a marked increase in ROS levels in the injured spinal cord at 3 days (Sham vs PBS vs EXO: 26.2 ± 1.572 vs 45.13 ± 1.25 vs 36.7 ± 1.251) (Fig. 5A, B) and 5 days(Sham vs PBS vs EXO:: 26.2 ± 1.572 vs 31.5 ± 0.624 vs 29.07 ± 1.115) (Fig. 5C, D) after SCI. Western blot results revealed that there was also a higher P-ERK/ERK level in the SCI group than in the sham group at 3 days (Sham vs PBS vs EXO: 0.506 ± 0.031 vs 0.903 ± 0.057 vs 0.677 ± 0.091) (Fig. 5E, F) and 5 days (Sham vs PBS vs EXO: 0.456 ± 0.022 vs 0.737 ± 0.138 vs 0.549 ± 0.052) (Fig. 5G, H) after SCI. It was also observed that P65 fluorescence intensity around the injury site was increased in the SCI group 3 days (Fig. 6A) and 5 days (Fig. 6B) after SCI. Hence, the above results suggested that SCI could activate the ROS-MAPK-NFκB P65 signaling pathway. Additionally, after treatment with DPSC-derived exosomes, increased ROS levels, P-ERK/ERK levels and P65 fluorescence intensity were all reduced compared with those of the PBS group (Figs. 5, 6A, B). Therefore, we learned that DPSC-derived exosomes can inhibit the ROS-MAPK-NFκB P65 signaling pathway in vivo. However, the ROS level, P-ERK/ERK level and P65 fluorescence intensity were still higher than those in the sham group.

### DPSC-derived exosomes can protect the injured spinal cord by inhibiting M1 macrophage polarization

At 3 days (Fig. 6A) and 5 days (Fig. 6B) after spinal cord injury, the CD86 fluorescence intensity was higher than that in the sham group, indicating more M1 macrophages around the injury site. After treatment with DPSC-derived exosomes, the number of M1-polarized macrophages around the injury site was less than that of the PBS-treated group and more than that of the sham group (Fig. 6).

**Fig. 7:**
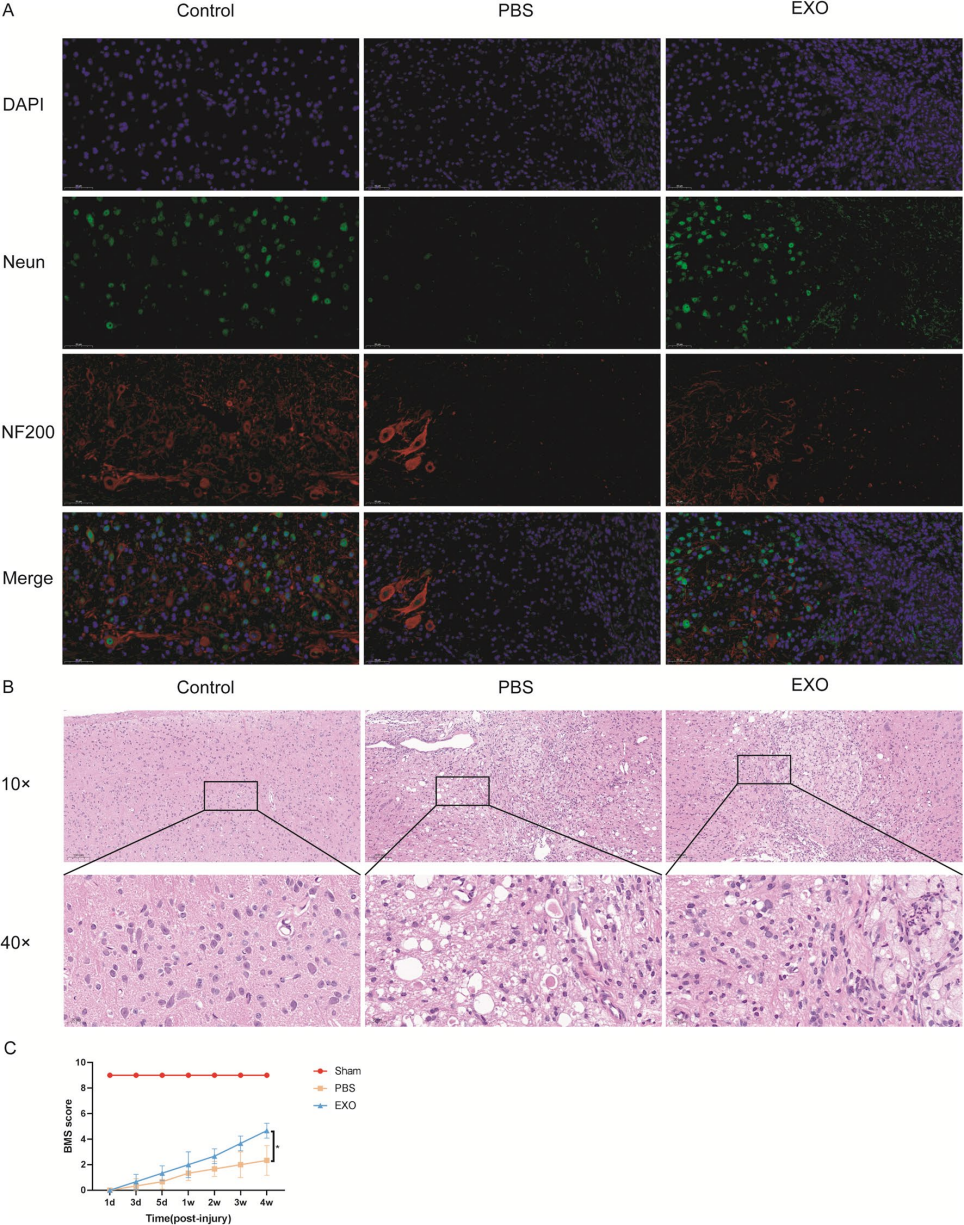
DPSC derived exosomes can protect injured spinal cord. **A** At 28 days after injured spinal cord, NF200 and Neun are determined by immunofuorescence in different groups. NF200 and Neun are selected as biomarkers of neurons. **B** Histological images (H&E staining) of longitudinal sections of injured spinal cords. There were transitional regions between the undamaged area and the scar area in the PBS-treated group. There were no or very few neurons in the transitional regions. The transitional regions were smaller or less obvious in the exosome-treated group. **C** BMS scores at diferent time-point after spinal cord injury. At 28 days after SCI, the exosome-treated group had higher BMS scores. *p < 0.05, ***p < 0.01

At 28 days after SCI, NF200 fluorescence and NEUN fluorescence were performed. The fluorescence results showed that there were transitional regions between the undamaged area and the scar area in the PBS-treated group (Fig. 7A). There were no or very few neurons in the transitional regions. The transitional regions were smaller or less obvious in the exosome-treated group. The same results were also found by HE staining (Fig. 7B). The results also showed that both the PBS- and exosome-treated groups presented complete hind limb paralysis with a BMS score of 0 at 1 day postinjury. At 28 days after SCI, the exosome-treated group had higher BMS scores (PBS vs EXO: 2.333 ± 1.155 vs 4.667 ± 0.577) (Fig. 7C).

## Discussion

**Fig. 8:**
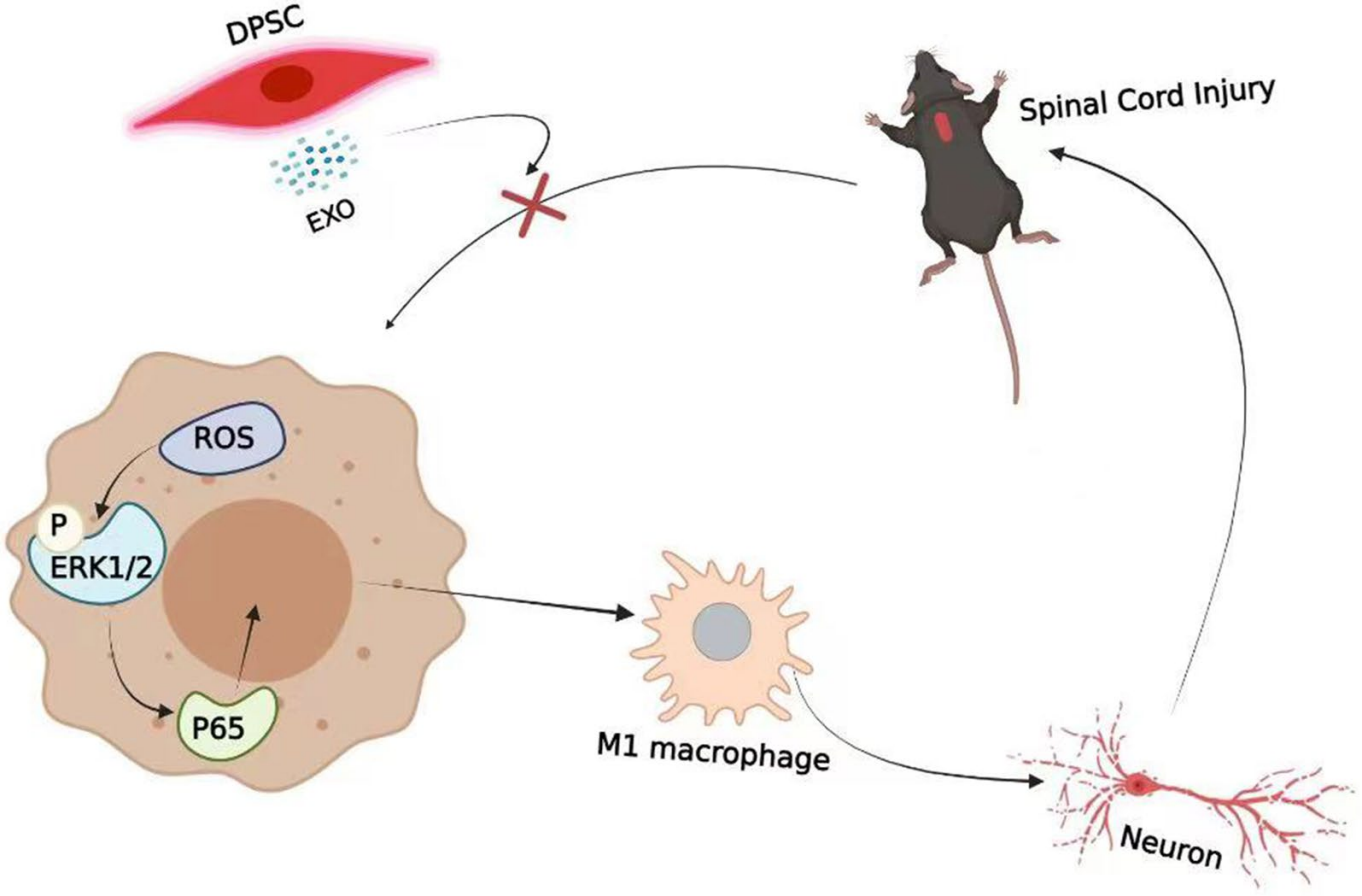
Schematic diagram showing the effects of dental pulp stem cells-derived exosomes on SCI

Most of the damage that occurs in spinal cord tissue after SCI is exacerbated by secondary damage [14, 48], and ROS play a key role in the secondary injury of SCI. Previous studies have demonstrated that ROS can activate the NLRP3 inflammasome, and inhibiting ROS production can reduce brain injury by downregulating NLRP3 [49–51]. Under normal conditions, the balance of ROS levels is regulated through ROS generation and inactivation. A lesion can break the balance by the depletion of antioxidant systems or the excess production of ROS, resulting in an increase in ROS levels [14]. ROS are commonly thought to be released from activated macrophages and neutrophils in the lesion site [52, 53]. Some studies have suggested that ROS may also trigger neutrophil-mediated inflammation, which is considered to contribute to secondary damage in spinal cord injury [17, 25]. However, the pathogenic role and mechanism of ROS in neuroinflammation are still not clear. A previous study reported that SCI is associated with inflammation by shifting the microglia/macrophage phenotype [54]. However, ROS levels were markedly increased within 2 h after injury [19] and occurred earlier than inflammation. We then hypothesized that ROS may trigger M1 macrophage polarization after SCI. The present study demonstrated that M0 macrophages can be shifted to the M1 phenotype after treatment with H_2_O_2_ for 24 h. The macrophage M1 polarization rate increased with increasing H_2_O_2_ concentration in the culture medium, suggesting that ROS concentration-dependent promotion of M1 macrophage polarization occurred. Hence, we also hypothesized that there was positive feedback between ROS and neuroinflammation after SCI, which may extend the inflammation period and the inflammatory regions. Disrupting the cycle may be a potential effective strategy to reduce secondary damage. Additionally, LPS was also used to treat cells to mimic cell damage. The results also showed that ROS levels were markedly increased within 2 h after culture with LPS. To investigate whether LPS activates M1 macrophage polarization mainly through ROS, quantitative RT–PCR analysis was performed to determine whether H_2_O_2_ upregulated the expression of iNOS, an M1 macrophage phenotype [44], earlier than LPS. Quantitative RT–PCR analysis revealed that both LPS and H_2_O_2_ upregulated the expression of iNOS at 6 h after treatment. This suggests that ROS may be just one of the ways LPS induces M1 macrophage polarization (see Fig. 8).

To support the specificity of the findings, we inhibited the production of ROS induced by LPS by DPI, which is a nonselective inhibitor of ROS-producing flavoenzymes. DPI markedly suppressed LPS-induced M1 macrophage polarization, suggesting that ROS play a key role in activating M1 macrophage polarization after lesion induction. Furthermore, we added H_2_O_2_ to the medium and found that DPI could not suppress the macrophage M1 polarization activation ability of H_2_O_2_ and that the ability of DPI to inhibit macrophage M1 polarization induced by LPS could be offset by H_2_O_2_. This result suggested that after injury, increased ROS can induce M1 macrophage polarization.

Activation of the MAPK signaling pathway plays an important role in the mediation of proinflammatory factors by microglia/macrophages after acute SCI [48]. MAPK is the best characterized oxidation–reduction sensitive signaling pathway, and the increase in ROS production could result in activation of the MAPK pathway [22, 26, 27], which could regulate inflammatory responses [48]. Studies have reported that activating the MAPK signaling pathway can lead to inflammation via downstream activation of the NFκB signaling pathway [55–59]. Hyperphosphorylation of MAPK molecules can activate NFκB release, and then NFκB is transferred into the nucleus, further activating inflammatory reactions. The NFκB signaling pathway is a master regulator of a vast repertoire of proinflammatory cytokines [60] and a ROS-sensitive signaling pathway [22]. Macrophages can be activated through the NFκB signaling pathway [61]. Macrophages are major innate immune cells and play a prominent role in the inflammatory process. Hence, we hypothesized that the ROS-MAPK-NFκB P65 signaling pathway may be a potentially targetable pathway for suppressing M1 macrophage polarization. To verify our hypothesis, the expression of t-ERK and p-ERK was determined using WB, and P65 was assessed by immunofluorescence staining. Western blot results showed that H_2_O_2_ could promote ERK phosphorylation, and DPI did not have an inhibitory effect on ERK phosphorylation caused by H_2_O_2_. Immunofluorescence staining results showed that H_2_O_2_ could also cause an increase in P65 fluorescence, while DPI could not effectively suppress an increase in P65 fluorescence. Interestingly, DPI effectively suppressed ERK phosphorylation and P65 immunofluorescence staining caused by LPS. Moreover, a P65 inhibitor suppressed macrophage M1 polarization caused by H_2_O_2_ and LPS. Additionally, an ERK inhibitor also reduced the macrophage M1 polarization rate and the P65 fluorescence increase caused by H_2_O_2_ and LPS. Therefore, these results proved our hypothesis that the ROS-MAPK-NFκB P65 signaling pathway was a potentially targetable pathway for suppressing M1 macrophage polarization.

Numerous studies have demonstrated that transplantation of mesenchymal stem cells (MSCs) is an ideal candidate for the cell-based treatment of SCI [62–64]. MSCs protect tissue damage mainly by suppressing inflammation through paracrine mechanisms and promote endogenous repair through stem cell regeneration and differentiation [10, 65, 66]. However, some drawbacks of transplanted stem cells, such as their lower survival rate, immune rejection, cell dedifferentiation and tumor formation, limit their application in treating SCI [10]. Exosomes are small paracrine particles with diameters ranging from 40 to 100 nm that are secreted by living cells and formed from proteins, signal proteins, cytoskeletal proteins, and growth factors [29–31]. In the present study, TRPS analysis revealed a diameter of approximately 100 nm. The expression of the exosome markers CD63, CD9, tubulin and the MSC marker CD73 in the DPSC-Exo samples and DPSC proteins was verified by western blot [67]. The protein marker albumin is negative in exosomes [67]. Stem cell-derived exosomes can exercise similar biological roles to stem cells. Additionally, exosomes are nanometer particles and can easily cross the blood–brain barrier (BBB) into spinal tissue, playing a therapeutic role. Many studies have demonstrated that stem cell-derived exosomes can reduce the ROS level in injured tissue and reduce M1 macrophage polarization in an SCI model [10, 36, 37]. Liu et al. reported that exosomes derived from mesenchymal stem cells could shift microglial M1/M2 polarization via miR-216a-5p [10]. Hong et al. reported that exosomes from adipose-derived stem cells could attenuate UVB-induced ROS [36]. Shen et al. reported that exosomes from adipose-derived stem cells could alleviate inflammation and oxidative stress by regulating the Nrf2/HO-1 axis in macrophages [37]. However, no study has investigated the relationship between increased ROS levels and M1 macrophage polarization in an SCI model. The present study demonstrated that ROS can induce M1 macrophage polarization. Hence, we hypothesized that exosomes may reduce M1 macrophage polarization through the ROS-MAPK-NFκB P65 signaling pathway in an SCI model. In vitro, DPSC-derived exosomes markedly reduced the ROS level and the M1 macrophage polarization rate of damaged cells. Western blot results showed that DPSC-derived exosomes could also inhibit ERK phosphorylation and P65 signaling pathway activation. To further confirm the findings, an SCI model was established. We observed that exosomes could markedly reduce ROS levels and the recruitment and invasion of M1 macrophages to lesions at 3 and 5 days after SCI, and activation of the ROS-MAPK-NFκB P65 signaling pathway was also suppressed. At 28 days after SCI, transitional regions between the undamaged area and the scar area were noted, and there were no or very few neurons in the transitional regions. Compared with the PBS-treated group, the transitional regions were smaller or less obvious in the exosome-treated group. The Basso Mouse Scale score of the exosome-treated group was significantly higher than that of the PBS-treated group. These results demonstrated that DPSC-derived exosomes could reduce neurological impairment by reducing macrophage M1 polarization through suppressing ROS-MAPK-NFkB P65 signaling pathway activation.

## Conclusion

In summary, this study revealed that the ROS-MAPK-NFkB P65 signaling pathway was a potentially targetable pathway for treating SCI. DPSC-derived exosomes could attenuate the inflammatory response and reduce neurological impairment by reducing macrophage M1 polarization through suppressing ROS-MAPK-NFkB P65 signaling pathway activation.

## Supplementary Information

** Additional file 1: Figure S1.** The surface molecule expression profiles and multilineage differentiation of MSCs. **A** Adipogenesis ability of DPSCs after induction. **B** Osteogenesis ability of DPSCs after induction. **C** The surface molecule expression of MSCs.

** Additional file 2: Figure S2.** **A** Higher H2O2 concentration (1 mMol/L) reached toxic level. **B** Both LPS and H2O2 can induce the expression of CD80 (A marker of M1 macrophages).
