# Supplementary figures and images for: Dental pulp stem cell-derived exosomes suppress M1 macrophage polarization through the ROS-MAPK-NFκB P65 signaling pathway after spinal cord injury
Source: J Nanobiotechnology. 2022 Feb 2;20:65. doi: 10.1186/s12951-022-01273-4 (PMC8811988; doi:10.1186/s12951-022-01273-4)

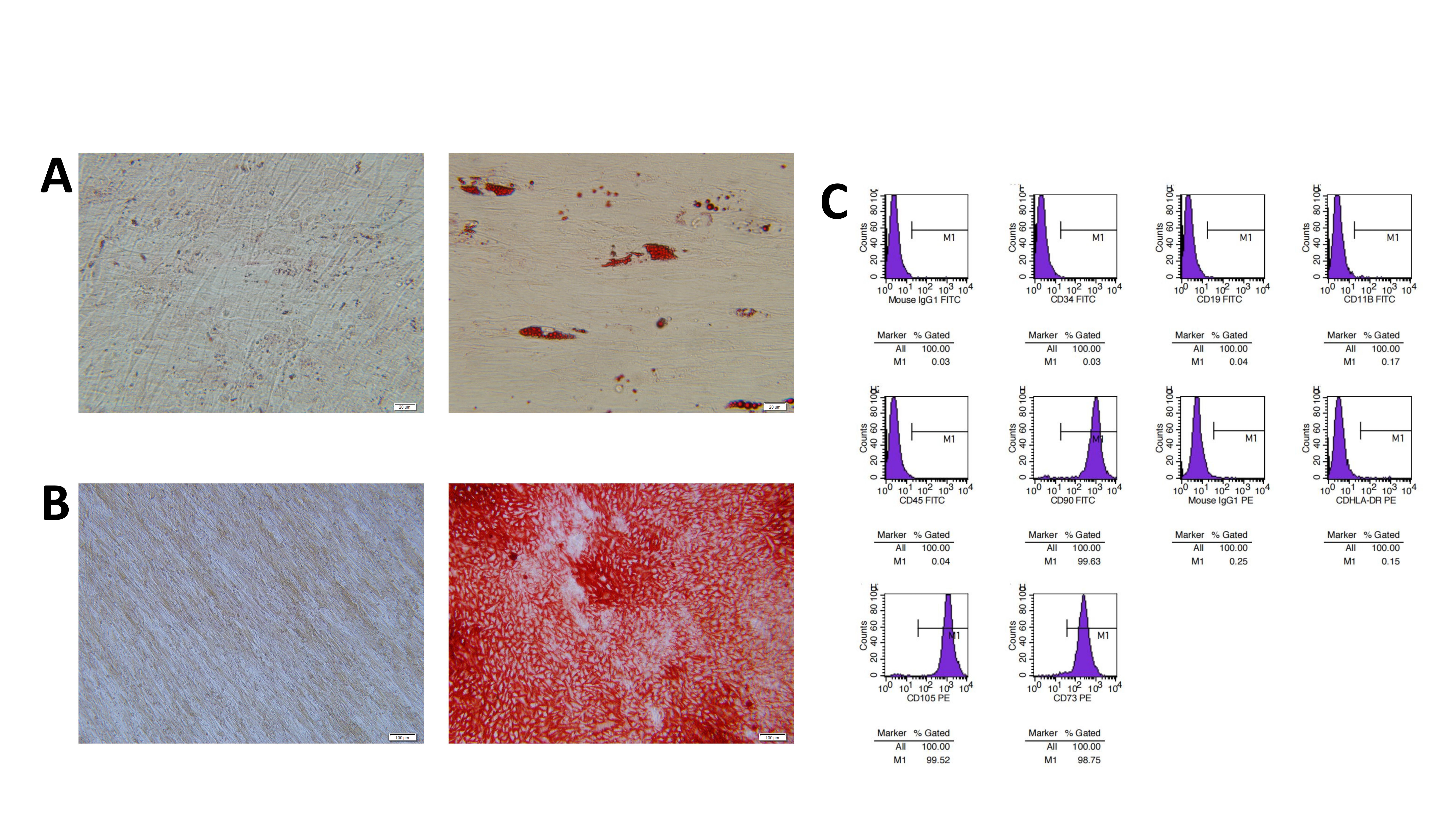

Supplement: Supplementary file 1 — Additional file 1: Figure S1. The surface molecule expression profiles and multilineage differentiation of MSCs. A Adipogenesis ability of DPSCs after induction. B Osteogenesis ability of DPSCs after induction. C The surface molecule expression of MSCs. [file 12951_2022_1273_MOESM1_ESM.jpg]

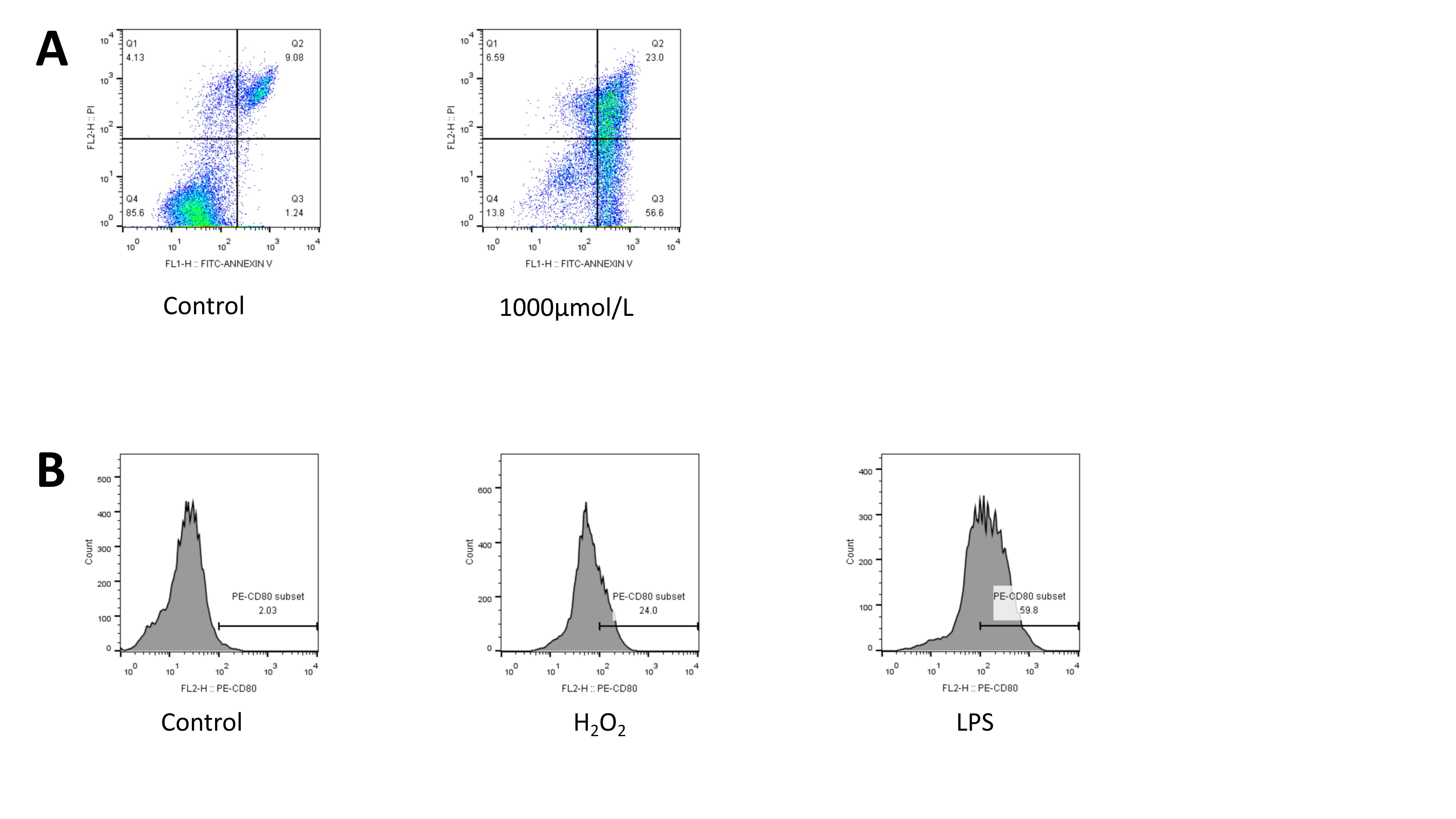

Supplement: Supplementary file 2 — Additional file 2: Figure S2. A Higher H2O2 concentration (1 mMol/L) reached toxic level. B Both LPS and H2O2 can induce the expression of CD80 (A marker of M1 macrophages). [file 12951_2022_1273_MOESM2_ESM.jpg]
